# Cyclin-dependent kinase 5, a node protein in diminished tauopathy: a systems biology approach

**DOI:** 10.3389/fnagi.2014.00232

**Published:** 2014-09-01

**Authors:** John F. Castro-Alvarez, S. Alejandro Uribe-Arias, Daniel Mejía-Raigosa, Gloria P. Cardona-Gómez

**Affiliations:** ^1^Neuroscience Group of Antioquia, Cellular and Molecular Neurobiology Area, Faculty of Medicine, University of Antioquia, Sede de Investigación UniversitariaMedellin, Colombia; ^2^Group of Biophysics, Faculty of Exact and Natural Sciences, Institute of Physics, University of AntioquiaMedellin, Colombia

**Keywords:** CDK5, tauopathy, GSK3β, phosphotases, Alzheimer's disease

## Abstract

Alzheimer's disease (AD) is the most common cause of dementia worldwide. One of the main pathological changes that occurs in AD is the intracellular accumulation of hyperphosphorylated Tau protein in neurons. Cyclin-dependent kinase 5 (CDK5) is one of the major kinases involved in Tau phosphorylation, directly phosphorylating various residues and simultaneously regulating various substrates such as kinases and phosphatases that influence Tau phosphorylation in a synergistic and antagonistic way. It remains unknown how the interaction between CDK5 and its substrates promotes Tau phosphorylation, and systemic approaches are needed that allow an analysis of all the proteins involved. In this review, the role of the CDK5 signaling pathway in Tau hyperphosphorylation is described, an *in silico* model of the CDK5 signaling pathway is presented. The relationship among these theoretical and computational models shows that the regulation of Tau phosphorylation by PP2A and glycogen synthase kinase 3β (GSK3β) is essential under basal conditions and also describes the leading role of CDK5 under excitotoxic conditions, where silencing of CDK5 can generate changes in these enzymes to reverse a pathological condition that simulates AD.

## Introduction

Neurodegenerative diseases are a growing problem in the elderly population. On such diseases is Alzheimer's disease (AD), the most common cause of dementia in people over the age of 65. Sporadic AD affects approximately 20 million people worldwide, and in Colombia, there are approximately 1000 carriers of the E280A mutation that causes early-onset AD (Lopera, 2002) that have been described in the department of Antioquia. AD is characterized by the abnormal folding and aggregation of the β amyloid (Aβ) and Tau proteins, which favor the formation of amyloid plaques and neurofibrillary tangles (NFTs), causing brain atrophy with marked cognitive impairment as the disease progresses (Jellinger, 2006; Querfurth and Laferla, 2010).

AD is characterized by two proteinopathies: amyloidopathy, a product of the abnormal cleavage of the amyloid precursor protein (APP), which leads to the accumulation of Aβ peptide in senile or amyloid plaques; and tauopathy, which is due to Tau hyperphosphorylation and aggregation. Tau is associated with microtubules (MTs) and, when it is abnormally phosphorylated, dissociates and aggregates, resulting in the formation of paired helical filaments (PHFs), which aggregate and form an insoluble conformation called NFTs. These two proteinopathies generate a loss of cell and tissue homeostasis during the neuropathological process of AD, which leads to progressive neuronal degeneration and the loss of the cognitive functions of the patient (Querfurth and Laferla, 2010).

Several studies have shown that normal Aβ processing is performed by a protease called α-secretase that cleaves APP to release a soluble extracellular fragment of 695 amino acids. The fragment that remains integrated in the membrane is processed by the action of a second enzyme, γ-secretase, releasing the carboxy-terminal part of the protein. This is known as the non-amyloidogenic pathway because the action of α-secretase prevents the formation of the Aβ peptide, preventing deposit formation (Jellinger, 2006; Querfurth and Laferla, 2010).

However, in the amyloidogenic pathway, one fragment of APP is processed differently. Another secretase, called β-secretase, cleaves APP, releasing a longer carboxy-terminal fragment, which releases the Aβ peptide after being processed by γ-secretase (Li and Sudhof, 2004). This peptide has limited solubility and forms auto-aggregates, which constitute the insoluble fibrils that are found in senile plaques. The actions of β-secretase and γ-secretase produce several types of Aβ peptides; the most common form is relatively soluble and has 40 amino acids (Aβ 40), whereas other forms that are less soluble have a length of 42 or 43 residues (Aβ 42–43) (Li and Sudhof, 2004).

The formation of NFTs due to the aggregation of amyloid plaques has been described in AD (Ingelsson, 2004). These structures are due to an abnormal increase in the phosphorylation of Tau associated with MTs and neurofilaments (NFs), preventing their assembly and stabilization, which is important for axonal growth and internal axonal transport (Avila et al., 2004). Tau is phosphorylated by different kinases that regulate its function and its binding to the neuronal cytoskeleton. Increased phosphorylation, known as hyperphosphorylation, may lead to a loss of function; avoiding the binding or detaching of Tau from MTs and causing its accumulation in the cytoplasm. This leads to the formation of PHFs, the extracellular accumulation of NFTs and subsequent brain damage (Grundke-Iqbal et al., 1986; Ingelsson, 2004).

In recent years, the central role of Aβ in AD has been questioned. It has been postulated that the production of Aβ peptide in quantities larger than normal is related to the origin of the disease and produces NFTs as a result of the damage inflicted by the presence of Aβ (Ferrari et al., 2003; Gotz et al., 2004). Another idea, based on the existence of dementia without senile plaques and dementia associated with parkinsonism linked to chromosome 17, or frontotemporal dementia, suggests that Tau hyperphosphorylation and the subsequent deposition of the protein is the main cause of neurodegeneration (Iqbal et al., 2005; Iqbal and Grundke-Iqbal, 2005). Although both postulates are part of the possible explanations for the phenomenon of neurodegeneration, only continued study of the particular context of AD will identify means of action and therapeutic possibilities.

Currently, much is known about the proteins associated with the development of tauopathy, but very little is known about the interaction between the set of proteins involved in Tau hyperphosphorylation and the subsequent development of tauopathy. Although the central importance of the kinases cyclin-dependent kinase 5 (CDK5) and glycogen synthase kinase 3β (GSK3β) and the protein phosphatases 1 (PP1) and 2A (PP2A) in this process has been described, it is not known with certainty how they interact and how this interaction contributes to Tau hyperphosphorylation in different states of kinase activation. This review aims to describe and model in an integrated manner the CDK5 kinase pathway as related to Tau hyperphosphorylation. This strategy outlines and groups isolated experimental data of the molecular species, or proteins, involved in CDK5 and Tau phosphorylation, describing the behavior of the signaling pathway in a pathological process such as AD.

## Systems biology

Systems biology (SB) is a scientific research approach that brings together knowledge from various disciplines to the study of living organisms from a systems perspective that assumes the component parts as a whole and the interactions between the components as their functional dynamics. Thus, cellular organization is described at different functional levels, ranging from the more specific intracellular processes (signaling pathways) to intercellular processes (neural networks). SB addresses the living organisms as an integrated and interacting network of genes, proteins, lipids, carbohydrates, and biochemical reactions from which life arises and persists (Yang et al., 2014).

The systems approach is not new, and many have worked to develop this idea. One of the most important developers was Bertalanffy, with his General Systems Theory published in 1969. In this paper, a system was defined as a set of objects united by some form of interaction or interdependence, displacing the idea that a system is only the sum of its components (Bertalanffy, 1969). The result was the study of living organisms as complex systems composed of elements that interact in complex ways and that emergent properties arise out of these interactions, which finally define the behavior of a system in a specific time and place (Yang et al., 2014).

Recently, SB has gained great interest in the scientific community due to the huge amount of data arising from the application of new high-throughput technologies and the inability of scientists to integrate and interpret this vast amount of information. From this point comes the need for approaches to integrate information and study the interaction between the molecular species identified; it is then when mathematical models arise as a possibility to simulate biological systems, integrating the data obtained experimentally and taking into account the interactions between the system components (Bowers and Boloure, 2001; Klipp et al., 2005).

The implementation of SB requires a process that can be defined in three basic steps. The first step involves the enumeration of the biological components involved in the process of interest, i.e., proteins, genes, lipids and other molecular species that are part of the system. The second step is to study the interactions of these components and generate a diagram that identifies system elements and their relationships; in this step, signaling pathways or routes of biochemical reactions are built. In the third step, these signaling pathways are reconstructed mathematically, creating computer models that emulate the behavior of the system, for analyzing, interpreting and predicting the functions of the phenomenon of interest (Palsson, 2006).

## First step: biological components

### Tau protein

The Tau protein was discovered in 1975 when it was found to be involved in the *in vitro* assembly of MTs added to tubulin (Murphy and Borisy, 1975; Weingarten et al., 1975). In humans, Tau is located on chromosome 17 and occupies approximately 100 kb with 16 exons. Tau localizes mainly to the brain, and 6 isoforms have been identified in the central nervous system, each of which contains two domains: the amino-terminal projection domain and the carboxy-terminal MT-binding domain. The projection domain is further divided into an acidic residue-rich region and a proline-rich region, and the MT-binding domain is divided into the basic region, tubulin-binding region and the acidic region (Avila et al., 2004; Hernandez et al., 2008).

Tau protein is easily phosphorylated, allowing the mobility of the protein within the neuron. When Tau is phosphorylated at proline-rich regions, it is distributed in somatodendritic compartments; when this region is dephosphorylated or when Tau is phosphorylated at its carboxy-terminal region, Tau is located in the distal part of axons (Mandell and Banker, 1996; Tashiro et al., 1997; Avila et al., 2004). Tau phosphorylation sites are divided into two types: those that can be modified by serine/threonine proline-directed kinases such as CDK5, GSK-3, mitogen-activated protein kinase (MAPK) and c-Jun N-terminal kinase (JNK), and non-proline-directed kinases such as MT affinity-regulating kinase (MARK), protein kinase A (PKA), protein kinase C (PKC), and Ca^2+^/calmodulin-dependent protein kinase II (CaMKII) (Trinczek et al., 1995; Reynolds et al., 2000; Avila et al., 2004; Wang et al., 2007; Yu et al., 2009). Depending on the phosphorylation pattern established by the kinases, different Tau functions are regulated in the cellular space.

The role of the Tau protein in the cell is directed by the establishment of MT dynamics and the stability of MTs in the cell (Witman et al., 1976; Trinczek et al., 1995; Tashiro et al., 1997). The function of Tau has been linked to the formation of cytoplasmic extensions, axonal transport and protection against deleterious compounds due to its association with MTs (Kosik and Finch, 1987; Lesort et al., 1997; Dawson et al., 2001; Johnson and Stoothoff, 2004). In Tau-deficient mice, a reduction in the number of MTs, small caliber axons, muscle spasms and behavioral deficits have been identified, although MT-associated protein 2 (MAP2) has been observed to partially compensate for the low Tau level (Dawson et al., 2001).

**Figure 1:**
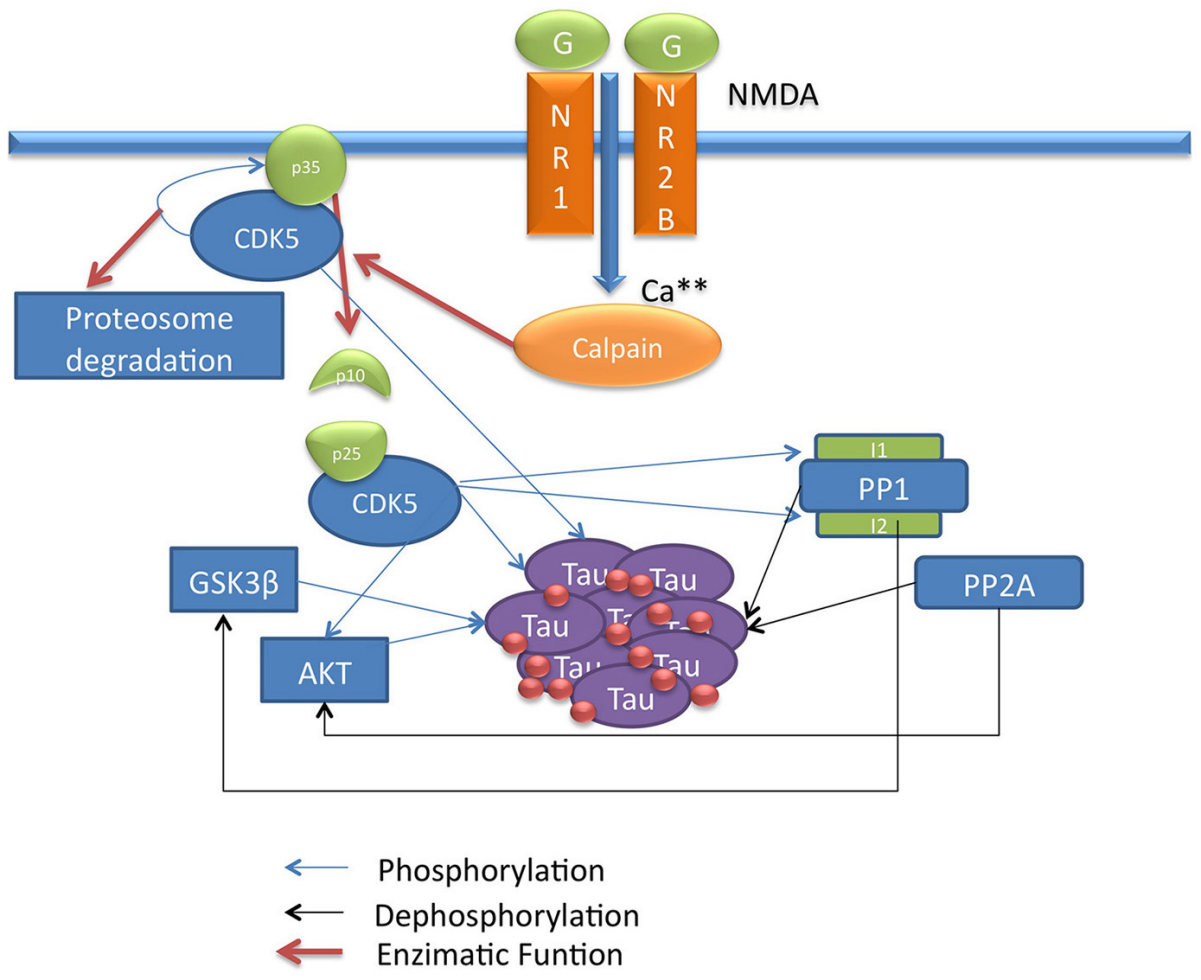
**Scheme of CDK5 signaling pathway involved in Tau phosphorylation**. This draw shows the CDK5 signaling pathway describing direct and indirect Tau phosphorylation by CDK5.

In AD and other tauopathies such as progressive supranuclear palsy (PSP) and frontotemporal dementia associated with parkinsonism linked to chromosome 17, a phenomenon of abnormal Tau hyperphosphorylation or phosphorylation is responsible for a set of alterations, such as axonal transport and mitochondrial and lysosomal dysfunction, among other functions associated with MTs, that can lead to neuronal degeneration (Avila et al., 2004; Iqbal et al., 2009). Upon the abnormal action of kinases and phosphatases, Tau dissociates from MTs and accumulates in the cytosol in packages of the abnormal PHFs, which in turn aggregate to form NFTs (Grundke-Iqbal et al., 1986; Anderton et al., 1995; Mandelkow et al., 2007) (Figure 1). The conditions that facilitate the aggregation and the formation of these structures are still unknown, but studies on the cellular context involved in this phenomenon are vitally important.

### CDK5

The family of cyclin-dependent kinases is a group of proteins involved in the regulation of DNA replication and cell division that control the transition from G1 to S phase and from G2 to M phase in the cell cycle. In humans, 10 members of the family have been described. Their function is regulated when a cyclin binds and activates a kinase, stimulating phosphorylation of its targets on serine/threonine-proline sequences. One member of this family is CDK5, which has 60% homology with CDK2 and has little involvement in the regulation of the cell cycle. CDK5 is highly conserved among species, and its expression is predominant in the nervous system. The function of CDK5 is not regulated by cyclins (Dhavan and Tsai, 2001; Ip and Tsai, 2008).

In the nervous system, CDK5 involvement has been described in neurite outgrowth, axonal guidance, neuronal migration, secretion, cytoskeletal dynamics, dopaminergic signaling, neuronal differentiation, synaptic transmission, neuronal survival, apoptosis and other functions that are still under study (Ip and Tsai, 2008). CDK5 also plays a role in molecular macro-complexes involved in intracellular transport, various signaling pathways and a large number of identified substrates that are related to the previously mentioned functions (Ip and Tsai, 2008). The majority of CDK5-deficient mice die *in utero*, and embryos that are born do not live more than 10 days postpartum. Post-mortem analyses show marked alteration in the formation of cortical, hippocampal and cerebellar layers, suggesting that CDK5 is essential for neuronal development (Ohshima et al., 1996; Fu et al., 2005).

CDK5 expression has been observed in different organs (Rosales and Lee, 2006), but the highest level of expression is found during brain development and in the adult nervous system. Its p35 and p39 activators have been found to be especially highly expressed in the brain, and although they are not cyclins and have very low homology with cyclins, their folded structure is quite similar to cyclin molecules involved in the cell cycle (Zheng et al., 1998; Ko et al., 2001; Kesavapany et al., 2003; Ip and Tsai, 2008). p35 and p39 have 57% homology with each other but exhibit different temporal and spatial expression patterns in developing and adult neurons (Zheng et al., 1998; Ko et al., 2001). Although individual deletion of p35 and p39 in mice does not duplicate the phenotype of CDK5-deficient animals, mice deficient for both activators do phenocopy CDK5 knockout; it is possible that the presence of one activator can compensate for the absence of the other (Ko et al., 2001).

Neuronal differentiation, which is characterized by dendritic growth, axon formation and the establishment of synapses, involves a wide variety of CDK5 substrates; these proteins modulate the cytoskeleton through actions on actin filaments, NFs and MTs to promote the development of the growth cone and neurite extension/retraction (Cheung and Ip, 2007). CDK5 inhibition in primary cortical cultures prevents neurite outgrowth; the involvement of p35 and p39 in growth cones and CDK5 association with members of the Rho/Rac GTPase (Ras-Homologous/Ras-related C3 Botulinum Toxin Substrate Guanosine Triphosphatase) family have also been reported (Nikolic et al., 1996; Ko et al., 2001; Angelo et al., 2006; Kawauchi et al., 2006). Of these family members, Rac binds to CDK5/p35 in a molecular complex that phosphorylates its effector PAK (p21-activated kinase), which regulates actin polymerization/depolymerization (Rashid et al., 2001).

The association of CDK5 with NFs and MTs has also been described; NFs are intermediate filaments associated with the dynamics of actin, MTs and axon formation. Moreover, MTs are tubulin polymers that, like NFs, maintain cell shape but are also associated with intracellular transport by the motor proteins dynein and kinesin. CDK5 regulates NF function, as well as MTs. CDK5 phosphorylates NFs directly and also phosphorylates MT-associated proteins such as MAP2, MAP1b, and Tau, which induces the formation and stability of MTs when phosphorylated. CDK5 also regulates the NUDEL protein, which is associated with dynein motor activity (Ohshima et al., 1996; Niethammer et al., 2000; Cicero and Herrup, 2005; Zheng et al., 2007).

The balance of CDK5 activation and its phosphorylation of different substrates is key for the proper performance of the neural network, synapses and for neuronal plasticity; changes that may affect this balance have been associated with pathological conditions such as AD, amyotrophic lateral sclerosis, Parkinson's disease and cerebral ischemia, among other disorders (Ip and Tsai, 2008). Imbalances in CDK5 are associated with alterations of the cytoskeleton, induction of apoptosis, increases in N-methyl-D-aspartic acid (NMDA) receptor-dependent calcium influx and other cell imbalances that can lead to hyperphosphorylation of different CDK5 substrates (O'hare et al., 2005; Plattner et al., 2006; Wen et al., 2007; Ip and Tsai, 2008; Querfurth and Laferla, 2010).

In AD, the relationship of CDK5 with Tau has been described; the CDK5/p35 or CDK5/p39 complex regulates the normal function of Tau in the cytoskeleton. Upon activation, CDK5 phosphorylates its p35 activator, and possibly p39, to induce proteasome-dependent degradation by regulating the duration of kinase activation (Hamdane et al., 2003; Iqbal et al., 2009; Peterson et al., 2010). In a pathological process such as AD, the activation of the calcium-dependent proteases m-calpain or μ-calpain can produce cleavage of the p35 and p39 activators, generating the p25 and p29 proteins, which are CDK5 activators with increased stability that induce a prolonged activation of CDK5 (Kerokoski et al., 2004; Peterson et al., 2010). This overactivation leads to hyperphosphorylation of different substrates, in this case the Tau protein, leading to PHF formation, the subsequent formation of NFTs and the disruption of the cytoskeleton and intracellular transport, inducing neurodegeneration (Ahlijanian et al., 2000; Hamdane et al., 2003) (**Figure 3**).

Despite the evidence linking CDK5 with Tau hyperphosphorylation, a consensus has not yet been reached on the role of CDK5 in the development of AD. However, there are some Tau phosphorylation sites for which CDK5 is known to be important, such as Thr181, Ser199, Ser202, Thr205, Thr212, Ser214, Thr217, Thr231, Ser235, Ser396, and Ser404, which are involved in the formation of PHFs (Gong et al., 2005; Wang et al., 2007). In addition, increased CDK5 immunoreactivity has been observed in neurons that begin the formation of NFTs compared with neurons that are part of mature NFTs (Gotz and Nitsch, 2001). Studies using compounds that promote the release of p25 and p29 by increased calcium influx, such as glutamate, NMDA, Aβ, and calcium ionophores, and those that describe the overexpression of p35, p25, and CDK5 show strong evidence for the involvement of CDK5 in Tau hyperphosphorylation and NFT formation (Ahlijanian et al., 2000; Kerokoski et al., 2002; Zheng et al., 2002, 2005; Hamdane et al., 2003; Plattner et al., 2006; Sengupta et al., 2006; Wang et al., 2007; Peterson et al., 2010). These studies suggest the involvement of CDK5 in AD, dependent on the cellular context, but without a doubt, CDK5 is one of the factors involved in its progression.

### Systems biology and tauopathy

SB approaches are currently being used to improve the understanding of AD. The main objectives of these studies are to model the interaction of genes and protein expression patterns and validate signaling pathways that are involved in the development of this disease. To this end, high-throughput techniques such as microarrays, which help to identify gene expression patterns in different stages of the disease, are used; proteomics approaches that provide knowledge of proteins that play a role in pathological processes and biomarkers of AD progression are also used. Mathematical models can integrate this information and are used to analyze signaling pathways where new therapeutic targets are being evaluated (Pasinetti and Hiller, 2008; V, 2009).

Tau has also been studied from the standpoint of SB; one study simulated the importance of Tau phosphorylation for MT assembly. This research concluded that different isoforms of Tau may have different conformations depending on the degree of phosphorylation and that these conformations influence the ability of Tau to bind to MTs. Furthermore, this study suggests that the phosphorylation pattern neutralizes the net positive charge of Tau residues, disfavoring their interaction with the negative charges of MTs that cause MT uncoupling and favoring the aggregation of Tau (Jho et al., 2010).

Some SB approaches have included CDK5 in mathematical modeling and have highlighted its importance in the function and regulation of other proteins. One model evaluated the regulation of phosphorylation sites of the dopamine- and cAMP-regulated neuronal phosphoprotein (DARPP-32) (Fernandez et al., 2006; Lindskog et al., 2006; Nakano et al., 2010; Qi et al., 2010). Other researchers have modified and supplemented this model by including new elements and conducting various *in silico* experiments (simulations), making this an ever closer approximation of the phenomenon that helps in better understanding the regulation of DARPP-32.

The DARPP-32 modeling included combinations of phosphorylation sites, its regulation by kinases and phosphatases and the influence of calcium and cAMP on these enzymes. The *in silico* experiments simulated changes in the levels of calcium and cAMP and the level and activity of each of the participating elements. The results from the different simulations were useful for understanding the influence of calcium and cAMP signals and the importance of each of the proteins included in the model. The variations in CDK5 activity and level showed that this kinase is a key player in the regulation of DARPP-32 and that the phosphorylation of DARPP-32 substantially affects its activation by inhibiting the action of PKA (Fernandez et al., 2006; Lindskog et al., 2006; Nakano et al., 2010; Qi et al., 2010).

The degree of Tau phosphorylation, the importance of phosphorylation in the aggregation of the protein and the influence of different enzymes on this process have been studied separately but without simultaneously taking into account the interactions among three phenomena. Thus far, this phosphorylation process and the influence of kinases such as CDK5 have not been described using an *in silico* model; this type of approach may aid in understanding the direct influence of CDK5 on Tau aggregation and the involvement of CDK5 in the regulation of substrates that mediate Tau phosphorylation.

## Step two: component interaction

To select and describe the components and interactions that are part of the CDK5 signaling pathway, it was necessary to perform a specialized information search using PubMed as a tool to access MEDLINE, the largest database of scientific literature that currently exists for biomedical sciences. Medical subject heading (MeSH) search terms, which allow a keyword search that is organized by subject headings, qualifiers (subheadings), definitions, synonyms and lists of closely related terms, were used to conduct this search. Of the retrieved documents, only original articles where the experimental design showed a direct biochemical connection with CDK5 and the substrate and that fit the description of a signaling pathway associated with Tau phosphorylation were selected. A description of the selected information is presented next.

### Role of CDK5 in tau hyperphosphorylation

Brain disorders caused by chronic conditions such as AD or acute disorders such as cerebral ischemia, among other pathologies, promote excitotoxicity or an alteration of cellular homeostasis that triggers an increase in glutamate concentration; glutamate binds and activates glutamate-dependent calcium receptors such as NMDA, α-amino-3-hydroxy-5-methyl-4-isoxazolepropionic acid (AMPA) and kainate receptors. When activated, these channels allow an influx of calcium and, in turn, promote the activation of calcium-dependent signaling pathways (Kerokoski et al., 2004). Excessive activation of these receptors may trigger the onset of pathological processes and even activate pathways involved in neuronal cell death (Lau and Tymianski, 2010). NMDA receptors are the most important of these receptors as they maintain activity for a longer period of time and allow higher calcium influx (Goll et al., 2003).

The influx of calcium into the cell activates calpain, a calcium-dependent thiol protease that cleaves the CDK5 activator p35 to p25 and p10 (Patrick et al., 1999). The p35/CDK5 complex phosphorylates p35 and becomes an auto-regulation signal that induces ubiquitination and the subsequent degradation of p35 through the proteasome (Patrick et al., 1998). Calpain cleavage eliminates this regulatory site so that p25 forms a more stable complex with CDK5, increasing its activity, which may lead to a long-term increase in Tau phosphorylation and neurodegeneration (Cruz et al., 2003). Moreover, the cleavage of p35 eliminates a myristoylation motif that anchors p35 to the membrane; this causes the p25/CDK5 complex to lose the peripheral membrane distribution of p35/CDK5 and allows the complex to access other types of substrates, suggesting that this process may be important for neurodegeneration (Ahlijanian et al., 2000) (Figure 1).

Phosphorylation of other substrates by CDK5 may indirectly promote Tau hyperphosphorylation. CDK5 has been described to play an important role in the activity of GSK3β, a serine/threonine kinase that is directly associated with Tau hyperphosphorylation (Mandelkow et al., 1992). Certain types of Tau phosphorylation by CDK5 increase GSK3β kinase activity for Tau; that is, the phosphorylation of Ser235 and 404 by CDK5 promotes an increase in GSK3β-mediated phosphorylation of Thr231 and Ser400 and 396, indicating that higher activity of CDK5 can generate increased Tau phosphorylation by GSK3β (Li et al., 2006) (Figure 1).

CDK5 can phosphorylate and promote the activation of epidermal growth factor (ErbB) receptors; when activated by the binding of neuregulin, a member of the family of epidermal growth factor proteins, ErbB receptors induce the phosphorylation and activation of protein kinase B (PKB) and AKT, a serine/threonine kinase that is involved in cell survival pathways (Li et al., 2003) and that inactivates GSK3β by phosphorylation at Ser9 (Cross et al., 1995; Wen et al., 2008). This indicates that CDK5 regulates GSK3β activity through different mechanisms and that CDK5 deregulation can promote the phosphorylation of Tau by GSK3β (Figure 1).

Phosphatases are another class of proteins involved in Tau activity; these proteins regulate the degree of Tau phosphorylation by dephosphorylating various residues. The most important phosphatases for Tau dephosphorylation are PP1 and PP2A, which show high activity for Tau (Liu et al., 2005). These phosphatases in turn regulate other substrates involved in the signaling pathway. Initially, PP1 activates GSK3β through dephosphorylation of Ser9 (Bennecib et al., 2000; Hernandez et al., 2010), and PP2A dephosphorylates and regulates AKT, inhibiting its activity on GSK3β (Mora et al., 2002; Resjo et al., 2002). Thus, phosphatases influence Tau phosphorylation through several mechanisms, and in a pathological condition such as AD where phosphatase activity is decreased (Liu et al., 2005), these enzymes are key factors in the development of the disease (Figure 1).

CDK5 is involved in the regulation of PP1 through phosphorylation of inhibitor-1 (I1) and inhibitor-2 (I2), two regulators that bind to PP1 and inhibit its activity (Oliver and Shenolikar, 1998). The dephosphorylated form of I2 binds to and inhibits PP1; CDK5 and GSK3β phosphorylate PP1 at Thr72, preventing I2 from acting upon PP1 (Agarwal-Mawal and Paudel, 2001). Unlike I2, the dephosphorylated form of I1 remains inactive and does not exert any function on PP1, whereas I1 phosphorylated at Thr35 and/or Ser67 by PKA and CDK5 binds to and inhibits PP1 (Huang and Paudel, 2000). In addition, CDK5 phosphorylates I1 at Ser6, which has no direct effect on the activity of I1; however, it has been described that I1 phosphorylated at Ser6 is less susceptible to dephosphorylation at Thr35 and thus remains active for a longer period of time (Nguyen et al., 2007) (Figure 1).

It is important to note that CDK5 is a kinase that plays a key role in regulating Tau phosphorylation either directly or by the regulation of other substrates. This signaling pathway describes a set of proteins and the relationships among them that support the influence of CDK5 on phosphorylated Tau; however, CDK5 and the described proteins have an interdependent network of relationships that we cannot cover in full using this theoretical and mathematical approach. To evaluate the participation of each of the proteins described in the signaling pathway, we confirmed the *in silico* analysis of the model by supporting our data and the relationship among the proteins in the signaling pathway.

## Step three: mathematical modeling

Mathematical modeling was performed to define the parts of the system and the way they interact with each other by means of mathematical equations. The model was described based on two types of reactions: binding, which can be described as the process of association and dissociation of two or more molecules, and enzymatic, where an enzyme binds to a substrate and converts it into a product. For the analysis of all components, ordinary differential equations were used that allow the description of the change in concentration of the different molecular species over time.

Once the molecular species and the reactions involved in the system (Table 1) were defined, the mathematical description of each of the reactions was performed separately according to the previously mentioned equations. Next, the coupling of the equations with common reactants was performed, which simplified the model because it consists of a large set of reactions occurring simultaneously. It is worth noting that the coupling of equations allows for any change in the concentration of the molecular species to interfere with the variations of other species. By way of example, the following reactions are illustrated:

**Table 1:** **Reactions and velocity constants**.

**Reations**	**K_**1**_ (μM^−**1**^**S**^−**1**^)**	**K_**_1**_ (**S**^−**1**^)**	**K_**2**_(**s**^−**1**^)**	**Source**
cal+ca↔cal.ca	1	20		Bhalla and Iyengar, 1999
cal.ca+p35↔cal.ca.p35→cal.ca+p25	0.12	0.44	0.11	Brenda
P35+CDk5↔p35.CDK5	20	1		Estimated
P25+CDk5↔p25.CDK5	20	0.1		Estimated
p35.CDk5+Tau↔p35.CDk5.Tau→p35.CDk5+pTau	0.007	0.17	0.043	Kerokoski et al., 2004
p25.CDK5+Tau↔p25.CDk5.Tau→p25.CDk5+pTau	0.04	0.88	0.22	Kerokoski et al., 2004
GSk3b+Tau↔GSk3b.Tau→GSK3b+pTau	0.012	0.24	0.06	Estimated
PP1+pTau↔pTau.PP1→Tau+PP1	0.01	0.08	0.02	Lau and Tymianski, 2010
pp2a+pTau↔pTau.PP2a→Tau+PP2a	0.017	0.16	0.04	Lau and Tymianski, 2010
GSk3b+pAKT↔GSk3b.pAKT→pGSk3b+pAKT	0.2	0.76	0.19	Brenda
PP1+pI1↔PP1.pI1	499.98	0.1		DOQCS
PP1+12↔PP1.12	200	1		Estimated
P35.CDK5+11↔P35.CDK5.11→P35.CDK5+pI1	0.3	7.6	1.9	Brenda
P25.CDK5+11↔P25.CDK5.11→P25.CDK5+pI1	0.6	8.9	2.3	Estimated
P35.CDK5+12↔P35.CDK5.12→P35.CDK5+pI2	0.05	0.8	0.2	Brenda
P25.CDK5+12↔P25.CDK5.12→P25.CDK5+pI2	0.082	1.12	0.28	Estimated
GSk3b+11↔GSK3b.11→GSK3b+pI1	0.047	0.72	0.18	Estimated
pl1 +PP2a↔p11.PP2a→PP2a+11	3.832	24	6	DOQCS
pGSK3b+PP1↔pGSK3b.PP1→GSK3b+PP1	25.56	55.2	13.8	Estimated
PP2A+pAKT↔PP2A.pAKt→PP2A+AKT	1.875	7.2	1.8	DOQCS
p25.cdk5+AKT↔p25.cdk5.Akt→p25.cdk5+pAkt	2.83	6.8	1.7	Estimated
p35.cdK5+Akt↔p35.cdk5.Akt→p35.cdk5+pAKt	1.15	4.1	1.0	Estimated
p35→	Ø	0.01	Ø	Estimated

*Descriptions of Reactions and velocity constants that form the model of the CDK5 signaling pathway involved in Tau phosphorylation*.

A+B → A.B
A+C → A.C → C+B

If each reaction occurs separately, the reactions will invariably end up forming B and A.B, and none of the species will influence the other. However, if these reactions occur simultaneously and in the same place, the result will be different because the change in the concentration of A depends on its binding to B and C; in reactions 1 and 2, the concentrations of C and B depend on other reactions that occur in 1 or 2. This example illustrates the interdependence that different molecular species may have, and therefore, it is necessary for these relationships to be reflected in the coupled differential equations.

After coupling the equations describing the reactions, a set of 40 ordinary coupled differential equations was built that was dependent on 62 reaction parameters or constants and 40 initial concentrations. The model was run at an initial concentration of 0.1 μM baseline calcium, and then a series of simulations were performed that consisted of variations in the concentration and reaction speed of the different molecular species. Initially, variations were made in calcium levels, the enzymatic activity of phosphatases and kinases was changed, the effect of various knockouts *in silico* (KOis) was tested, and *in silic*o silencing (SIS) of CDK5 and GSK3β was evaluated under different conditions.

The model and the simulations that were performed used calcium influx as input and the levels of unphosphorylated or phosphorylated Tau (pTau) as output, noting the ratio between the two. Specific Tau phosphorylation events were not taken into account because of difficulties with assigning constants that could account for a different phosphorylation pattern from normal; therefore, pTau is assumed to be an indicator of the level of phosphorylation, and increased pTau is regarded as a trend indicating hyperphosphorylation.

**Figure 2:**
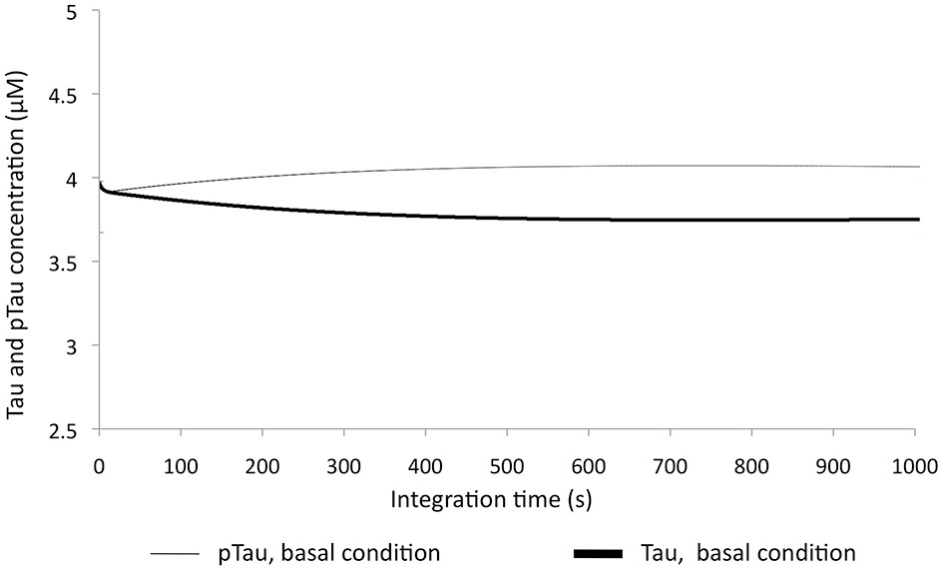
**Change in Tau and pTau levels at basal conditions**. There are stable behavior between two forms of Tau

Tau and pTau levels were studied at basal conditions; i.e., the model was run without any modification. It was found that Tau and pTau levels were balanced, indicating that approximately 50% of total Tau and pTau were constant (Figure 2). This suggested that there was a balance between the action of kinases and phosphatases, which is consistent with the known regulation of both types of enzymes, because Tau phosphorylation state depends on both the relationship between phosphatases and kinases and the role of these proteins in various pathological states such as AD (Wang et al., 2007; Morris et al., 2011).

**Figure 3:**
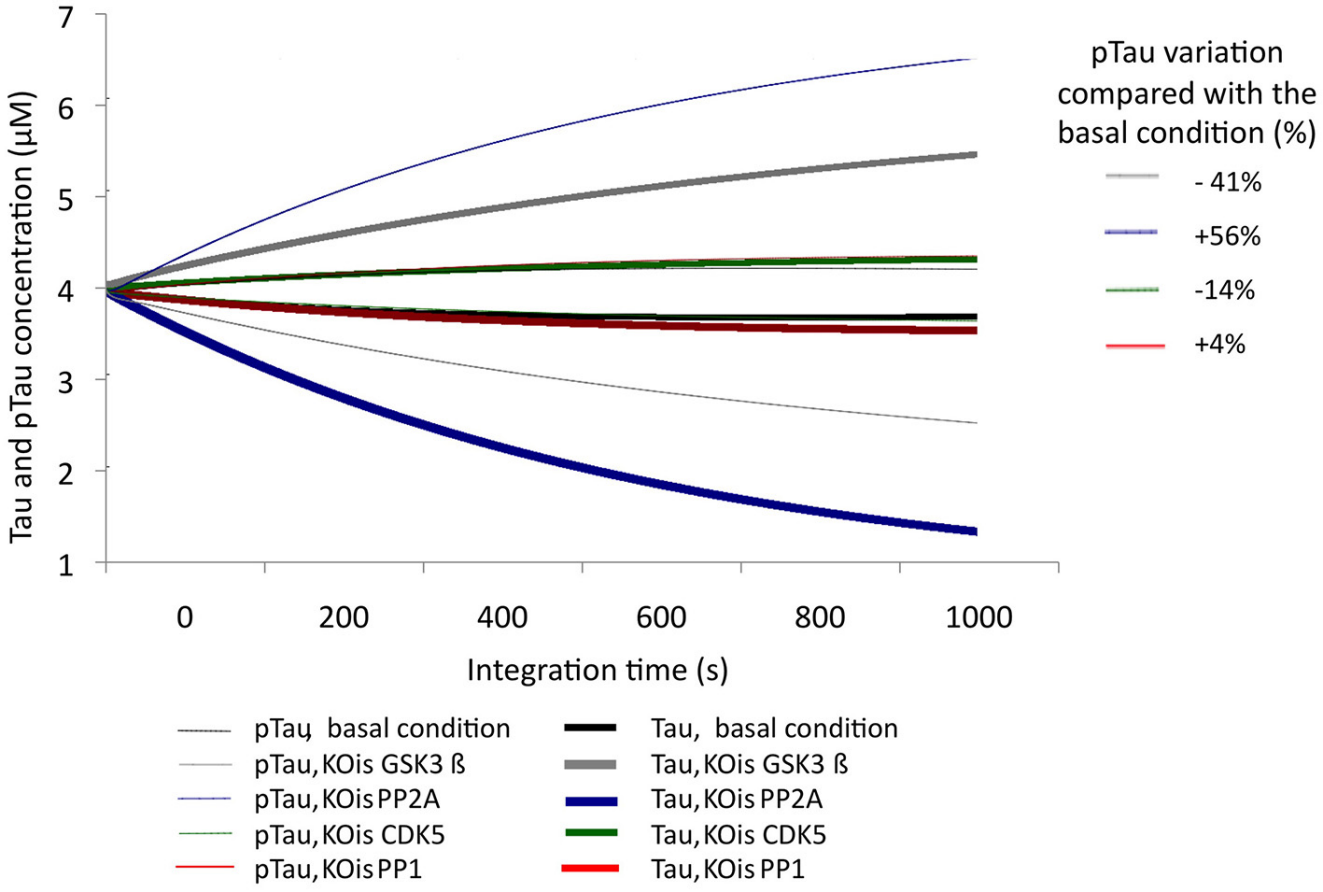
**Variation in the levels of Tau and pTau after KOis of CDK5, GSK3 β, PP2A, and PP1**. K2 was modified, which decreased this value by 100%. The inset in the figure indicates pTau variation compared with the basal condition.

To examine the sensitivity of the model to variations in different molecular species, KOis were performed that consisted of completely suppressing the activity of the molecular species by reducing the K2 constant (Vmax) to a number very close to zero. It was observed that the model was sensitive to dramatic changes in GSK3β, which decreased pTau levels to 40.5%, and changes in PP2A, which increased pTau levels to 56%. The KOis of CDK5 showed a change in pTau of 13%, and the KOis of PP1 did not alter pTau significantly (Figure 3). These results show that the system is more sensitive to proteins that have little regulation in our model; despite this, the model is stable to 100% variation because the maximum change generated only represented a 56% increase in pTau specifically with the KOis of PP2A.

**Figure 4:**
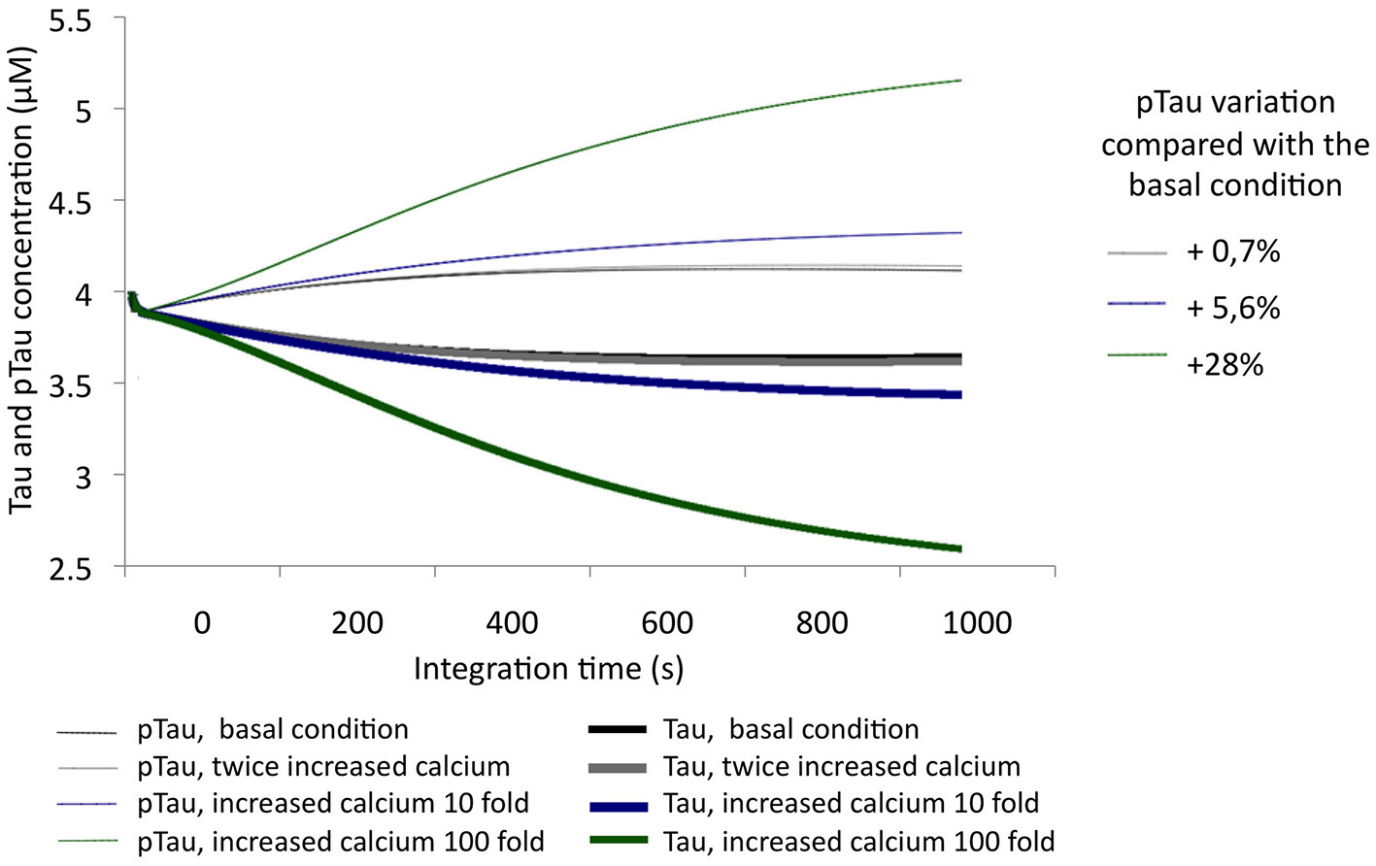
**Variation in the levels of Tau and pTau in response to variations in calcium levels**. Levels were increased 2-fold (0.2 μM), 10-fold (1 μM), and 100-fold (10 μM). The inset in the graph shows the relative increase of pTau compared with the basal condition.

After evaluating Tau and pTau levels at basal conditions, calcium levels were increased by 2-, 10-, and 100-fold from an initial concentration of 0.1 μM. Thus, an unbalanced condition of calcium homeostasis or an excitotoxic process, which is characteristic of pathological processes, was simulated. With increasing calcium concentration, pTau increased, reaching even a 28% increase over the initial condition (Figure 4). This indicates that calcium levels are a key factor in Tau phosphorylation; a significant increase in calcium influences the activity of CDK5 by activating calpains and promoting p25/CDK5 complex formation, which then has the ability to phosphorylate Tau over a longer period of time.

**Figure 5:**
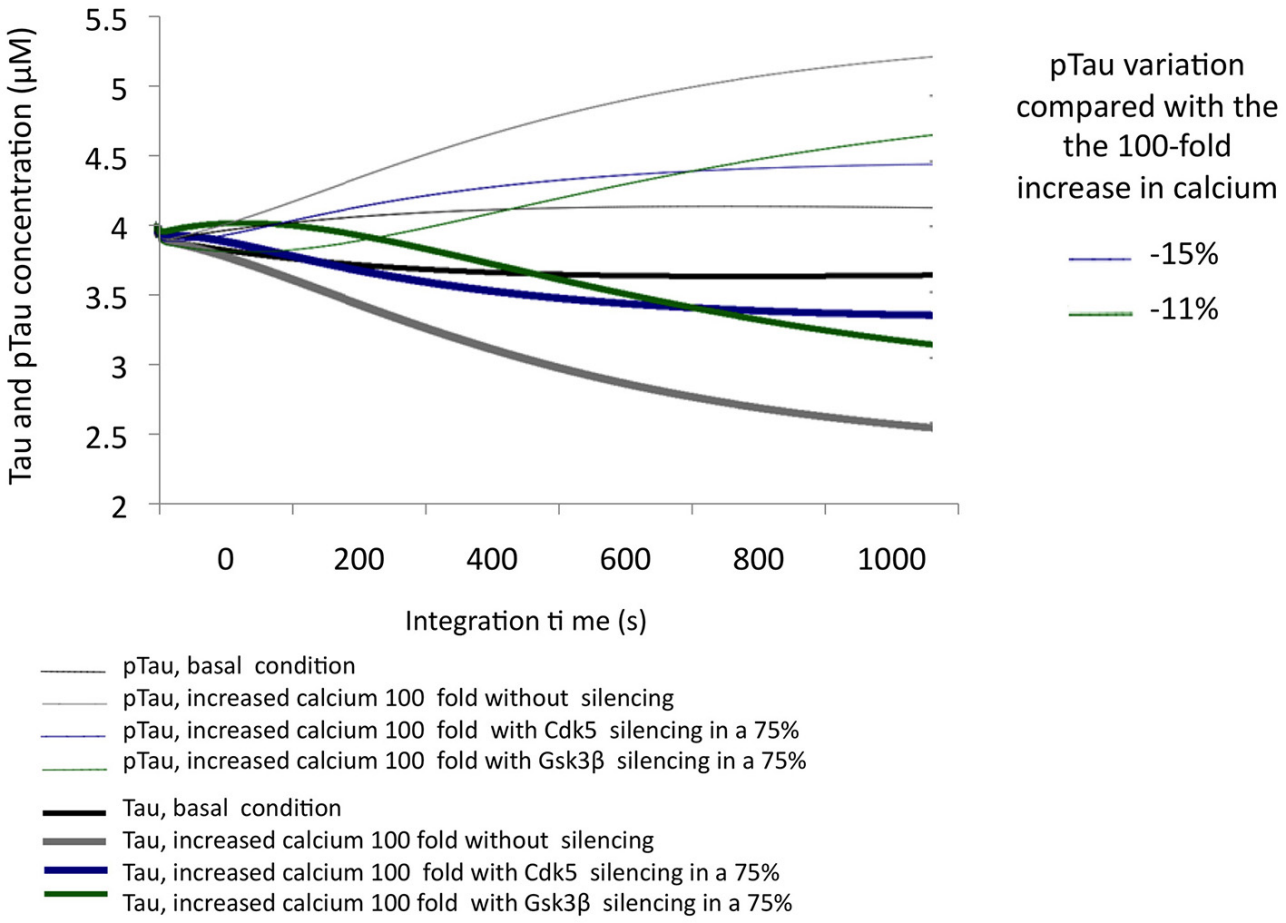
**Variation in the levels of Tau and pTau in response to the SIS of CDK5 and GSK3 β in conditions simulating excitotoxicity with a 100-fold increase in calcium**. CDK5 and GSK3β were silenced by 75%. The inset in the graph indicates the decrease in pTau in response to increased calcium.

The simulation of a 100-fold increase calcium added to SIS of GSK3β allowed the observation of the influence of this protein in a condition of excitotoxicity. A 75% decrease in the levels of GSK3β reduced pTau by 11%; although this is a significant reduction, we note that it is less than the 15% achieved by CDK5 reduction under the same conditions (Figure 5), unlike what was observed in the KOis under basal conditions (Figure 3). This result shows the importance of CDK5 and GSK3β and the importance of both in Tau phosphorylation under conditions of increased calcium levels.

**Figure 6:**
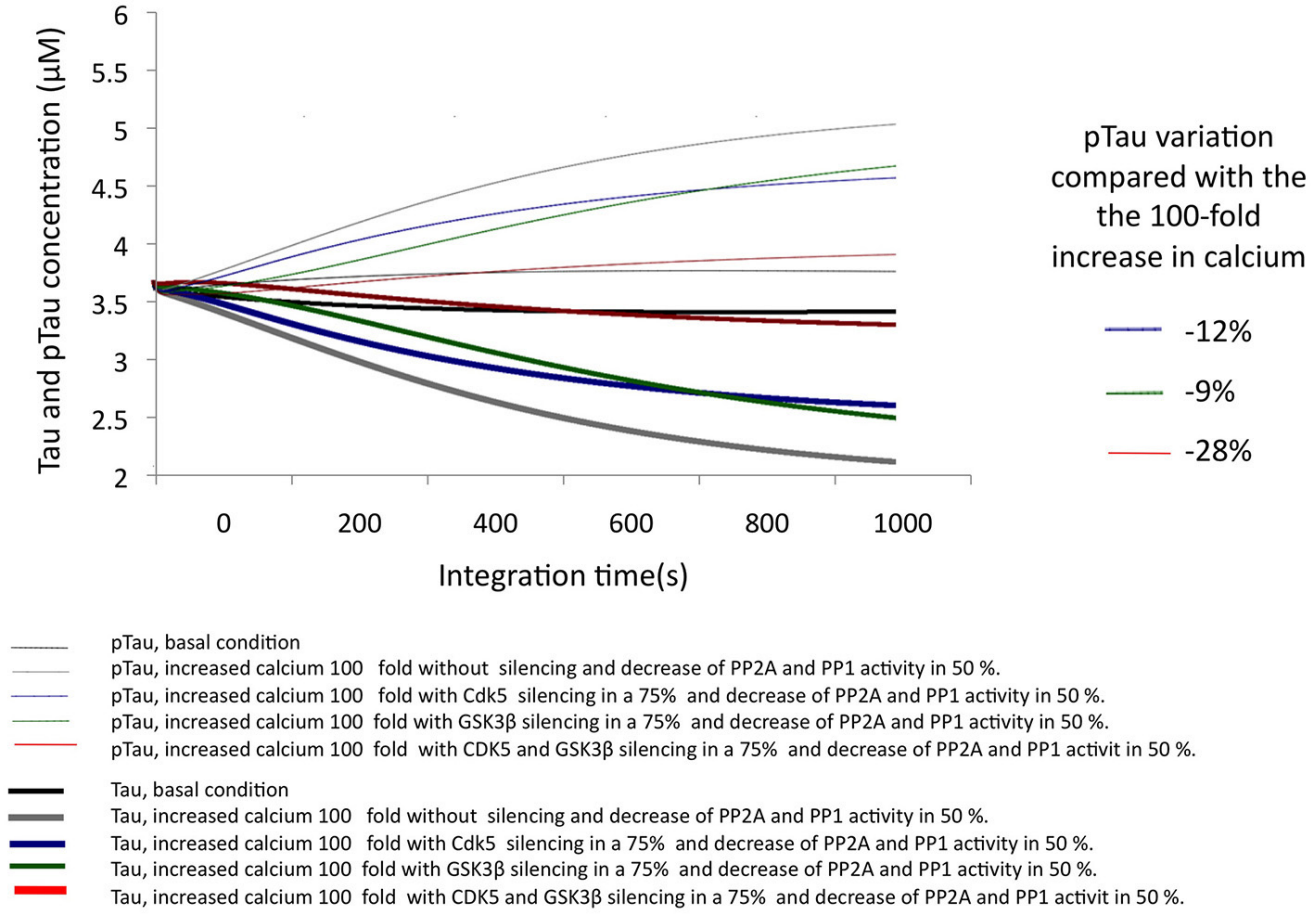
**Changes in the levels of Tau and pTau in response to SIS of CDK5 and GSK3 β in conditions simulating AD with increased calcium levels and a decrease in phosphatase activity**. CDK5 and GSK3β were silenced by 75%. The inset in the graph indicates the decrease in pTau in response to increased calcium.

In AD, it has been described that the excitotoxic process, as well as the increase in p25-mediated CDK5 activity and a decrease in the activity of phosphatases, plays a key role in the development of the disease. To simulate a similar condition, calcium concentration was increased by 100-fold, and the activity of both phosphatases was decreased by 50% (Figure 6). Under these conditions, the simulation of the pathological condition significantly increased pTau concentration (46%) (Figure 6). This strong trend toward hyperphosphorylation is consistent with the descriptions of AD where the presence of NFTs and Tau aggregation is a diagnostic criterion and is closely related to the pathogenesis of this dementia.

The SIS of CDK5 and GSK3β were assessed under the same conditions, simulating a pathological state. The SIS of CDK5 and GSK3β separately influenced pTau levels similarly, although CDK5 had a greater effect by decreasing pTau by 11.6%. In addition, the simultaneous silencing of both kinases produced a 28% decrease, reversing the effect caused by the pathological conditions (Figure 6); this suggests a synergic activity between depletion of the two kinases in decreasing pTau and emphasizes the importance of intervening in the regulation of both proteins in a disease process such as AD.

## Conclusions

**Figure 7:**
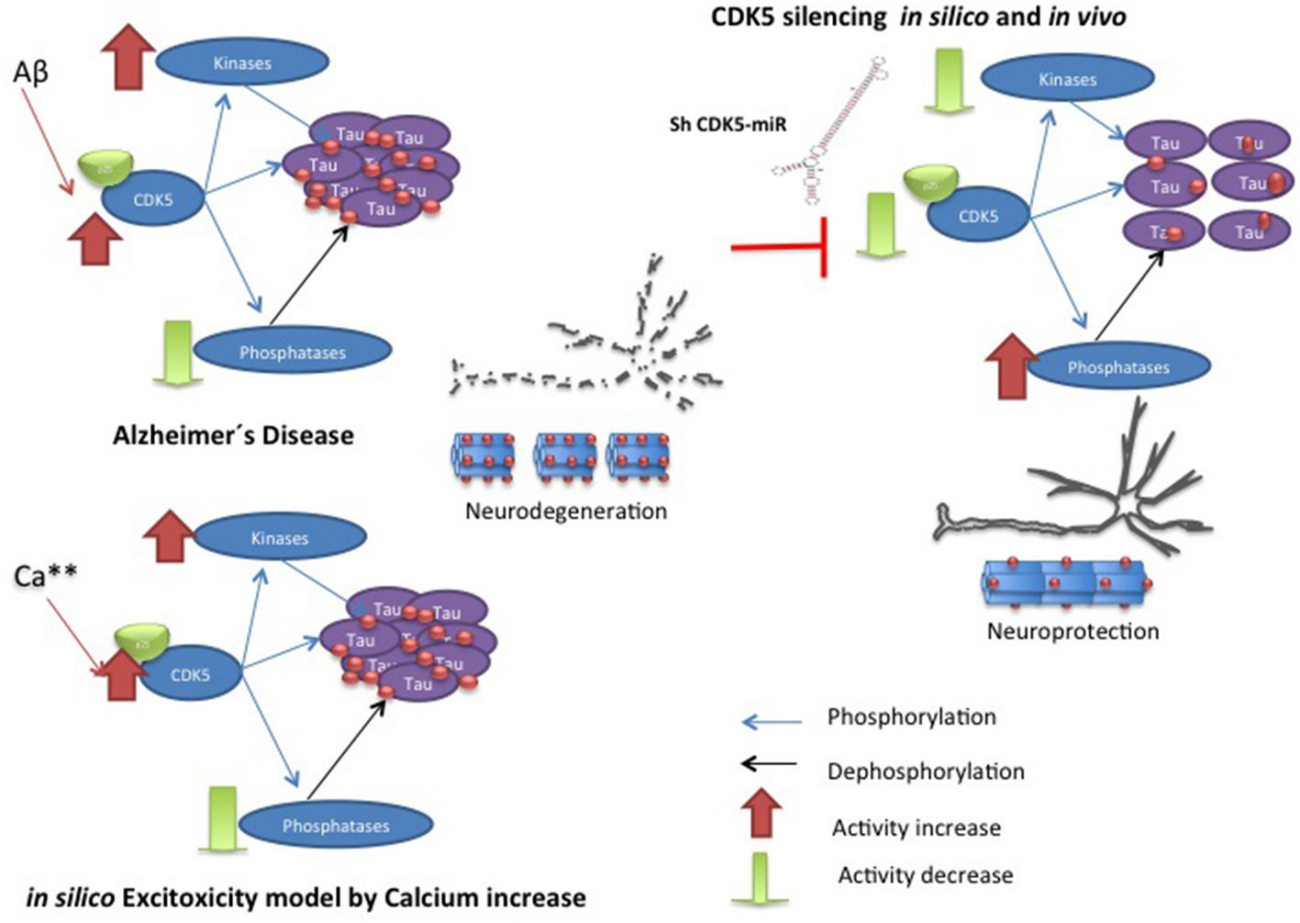
**Hypothetic model of CDK5 knockdown action on reversion of tauopathy in AD**. The toxic events in AD induce imbalance between kinases and phosphatases, where CDK5 overactivation produce hyperphospholylation of Tau and neurodegeneration. Our *in silico* model can simulate it, when the calcium increase induces more phosphorylation of Tau. However, CDK5 Silencing *in vivo* and *in silico* supported CDK5 as a node protein diminished tauophaty through reversion of GSK3 β upregulation and preventing reduction of phosphatases.

This review shows the dynamic complexity of a cellular system that is in constant action. The scientific literature provides largely isolated experimental data combined with theoretical data capable of providing a specific cellular context. SB integrates experimental data and creates computational contexts limited by existing data but that is dynamic, inclusive and is able to create new questions to gain an understanding of biological phenomena. In our previous experimental results show a chronic disease model and the behavior of the molecular species after the decrease of CDK5 (Piedrahita et al., 2010; Castro-Alvarez et al., Submitted). Although more experiments are needed to determine the causality of the results, the computational model and the experimental results show the importance of GSK3β and PP2A in conjunction with CDK5 in the process of Tau hyperphosphorylation (Figure 7).

Our present computational model approximated the conditions reported in the literature; thus, it was confirmed that the deregulation of CDK5 induced by excitotoxicity is directly involved in Tau hyperphosphorylation and that the inhibition of this kinase in a murine model prevents Tau hyperphosphorylation and aggregation in association with the described pathway. The work presented here indicates that PP2A is more important than PP1 for Tau dephosphorylation and that its action regulates phosphorylation events mediated by different kinases. In this case, inhibition of CDK5 may increase phosphatase activity in the context of a chronic disease, facilitating Tau dephosphorylation and aiding in the disappearance of Tau aggregates. Together with phosphatases, the synergistic role of CDK5 and GSK3β appears to be essential for tauopathy, and decreased CDK5 inhibits GSK3β function, which affects the disappearance of tauopathy.

Simulations using this model allowed the observation of the behavior of Tau and p-Tau at different calcium concentrations; however, it is important to describe in more detail the factors involved in the proposed signaling cascade to build a model that better approximates the chronic phenomenon of Tau hyperphosphorylation in AD. To this end, it is necessary to define in more detail the PP2A and GSK3β signaling pathways to improve the reproducibility of the model with other inputs. It is also essential to include new elements that may have a direct or indirect action on Tau phosphorylation and that are not associated with CDK5.

## Author contributions

John F. Castro-Alvarez, design and acquisition data, analysis and interpretation data, manuscript preparation; S. Alejandro Uribe-Arias, design and acquisition data, analysis and interpretation data, manuscript preparation; Daniel Mejía-Raigosa, design and acquisition data, analysis and interpretation data; Gloria P. Cardona-Gómez manuscript preparation and critical revision. All authors read and approved the final manuscript.

### Conflict of interest statement

The authors declare that the research was conducted in the absence of any commercial or financial relationships that could be construed as a potential conflict of interest.
